# Iranian nursing applicants' perception of the nursing profession: A qualitative study

**DOI:** 10.1002/nop2.1629

**Published:** 2023-01-29

**Authors:** Vahid Zamanzadeh, Akram Ghahramanian, Leila Valizadeh, Farzaneh Bagheriyeh

**Affiliations:** ^1^ Department of Medical Surgical Nursing, School of Nursing and Midwifery Shahid Beheshti University of Medical Sciences Tehran Iran; ^2^ Department of Medical Surgical Nursing, School of Nursing and Midwifery Tabriz University of Medical Sciences Tehran Iran; ^3^ Department of Pediatric Nursing, School of Nursing and Midwifery Shahid Beheshti University of Medical Sciences Tehran Iran; ^4^ Department of Medical Surgical Nursing, School of Nursing and Midwifery Urmia University of Medical Sciences Tehran Iran

**Keywords:** factors influential, nursing applicants, nursing profession, perception, qualitative study

## Abstract

**Aim:**

This study was conducted to describe the perceptions of nursing applicants about their chosen profession and to explore the factors which influenced their understanding.

**Design:**

A qualitative study with a conventional content analysis design.

**Methods:**

Participants were 19 nursing applicants enrolling in nursing schools in three provinces of western Iran. Data were collected using semi‐structured interviews. The content analysis of the interviews was done according to the steps proposed by Zhang and Wildemuth.

**Results:**

The mean age of participants was 20 years (SD = 2.5), and 11 of them (57.9%) were females. Participants understood the nature of nursing work to encompass only a limited level of independence; they viewed it as a feminine profession, and as a job with spiritual rewards. The perceived content of nursing work included providing help and patient care in the hospital with a focus on performing procedural tasks. Applicants' perceptions of nurses' characteristics included strong physical endurance, communication skills, emotional strength and low intellectual skills. The professional status of nursing was perceived simultaneously as having high job security but limited potential for career advancement and professional growth. The factors reported to influence applicants' perceptions were related to the media, academic–career counsellors, personal factors, and family and friends.

## 1 BACKGROUND

As the largest group of healthcare professionals, nurses have an essential role in promoting individual and community health (Smiley et al., 2018). However, a shortage of nurses has become a global problem in both developed and developing countries (Rankin, 2013). The World Health Organization (WHO) has predicted that the shortage of nurses will reach 12.9 million by 2035 (Arif & Khokhar, 2017; Gulland, 2013).

At present, a co‐incidence of supply and demand factors is driving the labour shortage (Drennan & Ross, 2019). The demand for nurses has increased due to the growing proportion of the population who are older people, while, simultaneously, supply has been limited by the retirement of nurses (Marć et al., 2019), a reflection of the ageing profile of the profession. One of the current major problems in the nursing profession is the high dropout rate of nursing students both during their studies and after graduation (Dall'Ora et al., 2020; Hamshire et al., 2019). Currently, about 15%–20% of nursing students per year drop out of school globally (Clements et al., 2016; Lunyk‐Child et al., 2001) resulting in considerable financial loss to taxpayers (Clements et al., 2016). The resulting staffing shortages can have unwanted effects on existing nursing staff (Jinks et al., 2014; Liaw et al., 2017) including decreased quality of nursing care (Alkaya et al., 2018; Kelly et al., 2021), burnout, reduced job satisfaction and increased turnover, especially among newly graduated nurses (Boamah & Laschinger, 2016; Drennan & Ross, 2019).

In Iran, after more than a century of professionalizing nursing, there is still absenteeism; new graduate nurses' intention to leave their job, nurses avoidance of introducing themselves in public communities and trying to introduce themselves in other professions (Hoseini‐Esfidarjani et al., 2018). Iranian nursing education costs a lot of money to train nurses; however, many students change their major or drop out of school, or some of them leave their jobs a few years after graduation (Shamsi & Peyravi, 2020). Many others remain ineffective in the profession. This issue leads to irreparable damage to public health and the country's economic resources (Neishabouri et al., 2018).

Perceptions of the nursing profession play an essential role in the retention of students in education and the retention of nurses in the nursing profession (Middleton et al., 2021). Studies indicate that the wrong image of the nursing profession is one of the reasons for the burnout of nursing students (Glerean et al., 2017). Mismatch between expectations and reality, and unmet expectations, causes nurses to leave the profession, especially newly graduated nurses (Flinkman, 2014). Misperceptions of the profession have been discovered since the 1990s and continue to persist (Hemsley‐Brown & Foskett, 1999). Several studies have agreed that choosing a career in nursing is often done with altruistic motives (Mooney et al., 2008).

Recruiting and retaining motivated students with a realistic understanding of the nursing profession has been suggested as one solution to address the nursing shortage (Kim et al., 2015). Nursing applicants' deliberate selection along with realistic understanding of the profession affect the students' satisfaction of education and success (Dall'Ora et al., 2020). Also, this could lead to their effective retention in the profession and also provides the ground for commitment to the profession (Swarna, 2015).

Based on a review study, nursing applicants' correct understanding of the profession at the job selection stage can be one of the ways to improve retention in the nursing profession (Glerean et al., 2019). However, there are very limited studies of nursing applicants' perceptions of the nursing profession. Studies have mainly focused on examining the change in nursing students' perceptions of the profession during nursing education (Ten Hoeve et al., 2017). In this way, the results of some studies indicate that education does not effect on changing nursing students' perceptions of the profession (Sand‐Jecklin & Schaffer, 2006; Toth et al., 1998). Considering the importance of identifying the perceptions of nursing applicants and the shortage of research on this field, this study was conducted to describe the perceptions of nursing applicants and factors influential on their understanding with a qualitative approach.

## 2 METHODS

### 2.1 Study design

Qualitative research seeks to explore and understand the inner world of individuals. Since experiences and perceptions are unique to each individual, the qualitative researcher can explore the meaning of phenomena from their point of view by entering into their world (Elstad et al., 2010). Considering that the research aimed to explore nursing applicants' perceptions of the nursing profession, a qualitative research method, conventional content analysis, was selected. This account of the study is based on the consolidated criteria for reporting qualitative studies (COREQ) (Tong et al., 2007).

### 2.2 Participants and setting

Participants in this study were 19 nursing applicants who were registering to enter nursing schools. They were selected using purposive sampling from among the nursing and midwifery schools in three major provinces in western Iran. When no new information was obtained, data were saturated after 17 interviews. Two additional interviews were conducted to confirm the data saturation. In the last two interviews, the data analysis led to the emergence of repetitive codes and no new code was obtained. Also, the decision to achieve data saturation was made through reviewing the codes and categories by the research team members and two experts other than the research team.

### 2.3 Data collection

Individual face‐to‐face interviews were used to collect data. Interviews began unstructured and gradually became semi‐structured interviews with the emergence of categories to gather more comprehensive information. When conducting the interview, the interview guide was used to ensure that all topics were stated. The interviews were conducted in person by previous arrangement with the participants in the nursing and midwifery schools.

Each interview lasted approximately 40–55 min. Before starting the interview, the purpose of the study, how to participate in the interview and recording the interviews were explained to the participants. After obtaining the informed consent, the interview began. At the beginning of each interview, after a few warm‐up questions, participants' demographic information was asked, and then the interview began with a general question. The main interview questions were: ‘How do you perceive the nursing profession?’ and ‘What factors contributed to your perception of nursing?’ Also, clarifying and exploratory questions such as ‘what does this mean?’, ‘can you give me an example?’ were asked to encourage the participants to describe real cases or situations. At the end of all the interviews, a few more open questions such as ‘Do you think there is something you did not say?’ were asked. Interviews continued until the data were saturated (i.e. no new themes were emerging from the analysis). All interviews were recorded using a voice recorder. Each participant was interviewed once. The data collection lasted approximately 7 months, and the entire study was conducted between May 2019 and April 2020 for 12 months.

### 2.4 Data analysis

Data were analysed using the conventional content analysis approach and according to the steps proposed by Zhang and Wildemuth (2009). The steps of this approach were conducted in the following order:

Step 1: Prepare the data

At this stage, the interviews were typed verbatim and entered into the qualitative data analysis software as a unit of analysis.

Step 2: Define the unit of analysis

The entire text of the interview was read several times so that the researcher was fully familiar with the data. Then they were coded by specifying meaningful units.

Step 3: Develop categories and a coding scheme

At this stage, the analysis units are categorized based on existing similarities and differences and the initial framework of the findings is formed. In this research, the constant comparative method was used to category semantic units. This method was used both for categorization and for merging similar category. In this research, no pattern or model was used for the initial categorization of data, but the codes formed separate categories after being placed next to similar codes, and these categories were also considered as the framework of the study. In other words, the data obtained from the interview were the source of the coding framework.

Step 4: Test coding scheme on a sample of text

In this study, the first interview was coded by the first and fourth authors of this study, the second and third interviews were coded separately by the first and second authors. Their coding was compared by the research team and due to the high similarity between the coders in the interviews, the coding process continued. Holisti's coefficient index was 0.85 and 0.79 for the first and second interviews respectively.

Step 5: Code all the text

After ensuring sufficient consistency in coding, coding rules were used in all parts of the text. Coding continued until new codes, subcategories and categories emerged.

Step 6: Assess coding consistency

In this study, after the completion of the coding of all the interviews, to ensure the absence of human factors such as fatigue and new impressions, one of the interviews was coded and compared separately by the second and fourth authors of this study. Also, during coding, the texts and codes were continuously reviewed by the research team members.

Step 7: Draw conclusions from the coded data

In this step, the compatibility of codes with categories and subcategories is examined and the obtained properties are compared with each other. The research team held several meetings and discussed and debated about the compatibility of codes with categories and subcategories.

Step 8: Report methods and findings

At this stage, the categories and subcategories were discovered and the relationships between them were identified.

### 2.5 Rigour

In this study, the criteria provided by Lincoln & Guba were used to ensure the trustworthiness of the data (Speziale et al., 2011). To ensure the credibility of the data, the researcher had a long‐term relationship with the participants, which aided trust and openness. During the study, interview transcripts, semantic units and extracted codes were presented to the participants to check their similarity with their experiences. Methods for determining dependency in this study included the researcher's long‐term involvement with the data and the peer review. Two external examiners with a doctorate in nursing who had qualitative research experience were asked to review the interviews, initial coding and categories. In this review, the cases on which there was disagreement were identified and discussed in a meeting. Finally, the closest code or category was selected with the agreement of the research team. About transferability, the characteristics of the research population and the research process were described clearly and accurately, to make it possible to follow the research path and key decisions made in the analysis. The conformability of the data were established by the researchers actively putting aside their thoughts and assumptions about the topic, accurate recording of the research procedure and documentation, refraining from the deep review of texts. This was all assisted by input from the rest of the research team.

## 3 RESULTS

The demographic characteristics of the participants have been summarized with mean and frequencies. The mean age of participants was 20 years (SD = 2.5), and 11 of them (57.89%) were females (Table 1). From the analysis of the interviews, 550 primary codes were extracted. After merging them, four categories and 11 subcategories were finally obtained. Data analysis revealed that the applicants' perception of the nursing profession was based on the nature and content of nursing work, nurses' characteristics and nursing job status (Table 2).

**TABLE 1:** Demographic characteristics of the participants.

Demographic characteristics	*N* (%) or mean (SD)
Gender	
Female	11 (57.9%)
Male	8 (42.1)
Age (years)	20 ± 2.5
Total number of participants	19

**TABLE 2:** Categories and subcategories resulting from the research.

Categories	Subcategories
Perceived nature of nursing work	Limited level of independence
	Professional femininity
	Work with spiritual reward
Content of nursing work	Helping and caring role in the hospital
	Procedure‐oriented
Characteristics of nurses	Strong physical endurance
	Communication skills
	Emotional strength
	Low intellectual abilities
The job status of nursing	High job security
	Low opportunity of career advancement and growth

The applicants understood the nature of nursing work with a limited level of independence, a feminine profession and a job with spiritual reward. The perceived content of nursing work included providing care and patient care in the hospital with a focus on performing procedural tasks. Applicants ‘perceptions of nurses’ characteristics included strong physical endurance, communication skills, emotional strength and low intellectual skills. The job status of nursing was also perceived with high job security and at the same time the opportunity of limited career advancement and growth.

### 3.1 Perceived nature of nursing work

Nursing applicants described the nature of nursing work with ‘limited level of independence’, ‘professional femininity’ and ‘work with spiritual reward’.

They believed that all nursing work is done according to specific instructions and protocols. In their view, nurses monitor the patient's condition in accordance with physicians' opinions and make decisions in very limited cases. Applicants emphasis was that physicians make key decisions about patient care and recovery.

What I have seen in the film and reality is that nurses rely on the doctor's statements, and they follow their decisions. The doctor orders, and the nurse executes. In addition, their work is based on a series of rules and guidelines. (Participant 12)

Nursing applicants considered nursing as a female job and more in line with female characteristics. In their view, women's emotional abilities in nursing work are beneficial.

Patient care requires strong feelings, kindness, and forgiveness that are more common in women, the school counselor told me, and I believe that. I mostly saw nurses as women because women do much better care than men. (Participant 14)

Nursing applicants said the nursing profession with a spiritual reward. They believed that nursing work could have other positive aspects besides salaries and financial benefits, such as the pleasure of helping others with all their being and caring for them.

The opportunity for humanity is great in this job. Helping and caring for a person is one of the most valuable tasks of a nurse. (Participant 8)

### 3.2 Content of nursing work

Nursing applicants' perceptions of the content of nursing work were identified in the two categories of ‘helping and caring role in the hospital’ and ‘procedure‐oriented’.

Nursing applicants stated that patients' care is one of the main duties of nurses. Helping and caring for patients were perceived as following the doctor's instructions and staying with the patient. They described nursing concerning the patient and the hospital, and none of them mentioned any role of the nurse about the healthy client or the community. They imagined the nurse's workplace only in the hospital, and they used the word patient in all their talks.

The workplace of a nurse is in the hospitals and clinics in which they take care of the patients according to the doctor's instructions. Their main job is to help and care for the patients. (Participant 3)

Most of the applicants did not have a picture of the current working conditions of nursing in their minds and they described the nurse's work as procedure‐oriented. Among the nursing procedures, they mentioned only injecting ampoules or IV and giving medicine.

Nurses mostly do the tasks such as giving medicine, injecting ampoules, wound dressing, and controlling vital signs. We, also, should be able to do these tasks later to be known as a good nurse. (Participant 12)

### 3.3 Characteristics of nurses

The nursing applicants described the nurses with characteristics such as ‘strong physical endurance,’ ‘communication skills’, ‘emotional strength’ and ‘low intellectual abilities’.

The applicants believed that the nurses must have strong physical endurance to perform their duties. The most important characteristics perceived in this subcategory were not to get tired of doing work quickly and the ability to do a lot of work during the day.

Sometimes that I went to the hospital, I saw that nurses always run this way and that way. Usually, the nurses stand for a long time throughout their shifts; sometimes, they carry heavy equipment. Also, the workload is high, so they need high physical strength to help their patients and not to get tired quickly. (Participant 4)

Applicants described the nurses' communication skills as listening to the patient's complaints, the ability to communicate with patients and family and the skill of empathy with patients.

Nurses are in contact with the patients continuously. They must be able to listen well to their talks. They must have good public relations to be able to adapt to different types of people. Patients are individuals who are hurt, and they must be able to empathize with them and understand them. (Participant 16)

Most participants described emotional strength as one of the important characteristics of the nurses. In their viewpoint, nursing is a stressful and busy job in which employees constantly face stress, death, suffering and pain of the patients. They must be able to control their emotions in painful situations.

The nurse must be emotionally strong because, in this major, she/he can witness the death of a human being at any time; she/he might take care of the patients who are in a lot of pain. (Participant 11)

From the participants' point of view, nurses need low intellectual abilities to perform their duties. They described nursing as a practical occupation with minimal need to use intellectual abilities.

This is mostly a practical major than an intellectual one. I am better in practical skills than sitting down and thinking about or analyzing a subject. (Participant 5)

### 3.4 The job status of nursing

Participants perceived nursing as a major with high job security and, at the same time, the low opportunity of career advancement and growth. Most of the applicants were interested in entering the field due to the availability of jobs and the assurance of employment.

I chose to nurse for my bachelor's degree because it has a job market, and I would be employed quickly. The career prospect of this major in our country is better than the other undergraduate majors, so I chose it. At least I am no more concerned about being unemployed. (Participant 9)

That's right that I want to study this major; however, I know that the possibility of growth is low here. (Participant 17)

### 3.5 Factors influential on the applicants' perception of the nursing profession

Nursing applicants described the factors influential on their perception of nursing in four categories: media, academic and career counsellors, personal factors, family and friends.

#### 3.5.1 Media

During the interviews, the nursing applicants stated that they obtained information about nursing through media such as TV movies, news, searching on the Internet and social networks such as telegram and Instagram. Participants mainly stated that they searched the Internet to choose this field, and they obtained information about it through various websites. They got different contradictory information through these websites.

Iran movies always show the nurses inferior to a doctor; someone who is injecting or writing. I changed my mind a lot when I saw the foreign movie “the nurses”. In this film, they have shown the nurses scientific and professional individuals. (Participant 11)

#### 3.5.2 Academic and career counsellors

Nursing applicants stated obtaining information from academic and career counsellors as a way to understand this major. During the interviews, it was found that the majority of counsellors did not have a positive view towards nursing; however, they emphasized more on the job security of the nursing profession.

The job counselor told me that nursing is a routine job and taking care of others. The only advantage is that you would be employed after graduation. (Participant 17)

#### 3.5.3 Personal factors

‘Interest in caring for and helping others’ and ‘hospitalization experience or presence in hospital’ were among the factors influential on applicants' perception of the nursing profession.

Applicants' interest in caring for and helping others was a factor in seeking information to better understand the major.

I had an innate interest in caring for and helping others, and that is why I sought information in this major. (Participant 6)

Participants stated the experience of hospitalization and presence in the hospital as a factor to better understand the nursing profession. They were also familiarized with the characteristics of nurses and the content of nursing work during their presence or hospitalization in the hospital.

I had an appendectomy during high school. While I was in the hospital, I saw how hard the nurses work, they are wonderful people, and that sparked me to think about entering this field. (Participant 2)

#### 3.5.4 Family and friends

The family was influential on the applicants' perception of the profession. Many participants reported that although their parents did not agree quietly with their child's education in nursing, they encouraged them to enter the field by informing their child about the job security of nursing.

My parents said: study this field now and let's see what happens, at least nursing has employment outlook. So in the future, you won't be unemployed and I chose this field because of this advantage. (Participant 8)

Friends were reported as one source of information in understanding the nursing profession. During the interviews, the participants stated that their friends imagined nursing as an occupation with low social level. Mostly, entering the field was not supported by friends.

I chose the nursing major, despite the fact that my friends did not like it and they disdained, in terms of class and such things, they said it is low class. They even told me not to choose the nursing, or consider it as your last choice. (Participant 12)

## 4 DISCUSSION

The purpose of this study was to describe the nursing applicants' perceptions of nursing and the factors influential on their understanding. Findings indicated that despite more than a century of professionalizing the nursing field in Iran, there is still no real understanding of this field among the nursing applicants. Applicants generally enter nursing education with a stereotypical and inaccurate picture of this major. What is important is that despite the old image of the actual nursing work and the description of the field as unattractive, the applicants were inclined to enter this major. The entrance of disinterested people with negative perceptions in nursing can have very adverse effects on the future of the nursing profession.

Findings showed that the applicants understood the nature of this profession as a feminine job. Despite the fact that nowadays many men have entered the nursing profession, gender attitude has not been separated from the nursing profession. The results of various studies are in line with the findings of this study (Arif & Khokhar, 2017; Dos Santos, 2020; Yi & Keogh, 2016). Gender as an invisible factor has caused the role and presence of men in the nursing profession to be low, despite the obvious and hidden organizational needs. Many men enter the profession without sufficient interest and motivation (Norman, 2015). The lack of independence of the nursing profession was another perception of nursing applicants. According to research findings, one of the causes of the low level of independence in nursing is the low social image of the profession and the ambiguity of nursing roles in society (Abrahamsen, 2019; Haron et al., 2014). Clarification and expansion of nursing roles at the community level is a great opportunity for nurses to present their abilities and capabilities to the community (Glerean et al., 2017; López‐Verdugo et al., 2021). The promotion of basic services by nurses will create a place to introduce the field of nursing to the public (Kim et al., 2015). Another finding of this study is that the field of nursing was perceived as a valuable profession relying only on spiritual values (Koo & Lin, 2016; López‐Verdugo et al., 2021). Academic nature of the field has not yet been recognized among the applicants (López‐Verdugo et al., 2021). Also, in many studies, the society's negative perception of the nursing profession is related to low academic standards (Grinberg & Sela, 2022; Liaw et al., 2017).

The applicants described the content of the nursing work as helping and caring for patients in the hospital and procedure‐oriented. Findings indicate that the applicants perceived the nurses' roles and responsibilities as incomplete and negative. In many studies, people's imagination of the nurses' role is limited to patient care in the hospital and performing repetitive procedures (Glerean et al., 2017; ten Hoeve et al., 2017). Applicants' perception of the nursing care role has been unprofessional and without the need for academic education, which is confused with the work of support staff. The need for knowledge and specialized skills in the nursing profession has been forgotten (Liaw et al., 2017; ten Hoeve et al., 2014). Therefore, it is very important that nursing education and healthcare institutions highlight the roles and duties of nurses clearly and unambiguously for the applicants.

Applicants described nurses with strong physical endurance, a need for communication skills, emotional strength and low intellectual skills. Also, in the study by Glerean et al. (2019), the applicants had similar perception of the characteristics of nurses in line with the findings of this study. In some studies, nurses have been described as kind, compassionate, highly perceptive and hardworking, but with limited intellectual abilities (Glerean et al., 2019; Norman, 2015; Straughair, 2012). It is very important for the applicants to have sufficient and correct understanding of the other characteristics of nurses, including their intellectual abilities. Unfortunately, the need for this ability to improve the quality of nursing care has been neglected (Pitt et al., 2015). Cognitive preparation is necessary for individuals to succeed in the theoretical and clinical practice courses (McNelis et al., 2010; Vierula et al., 2020). Nurses' cognitive abilities play a key role in problem‐solving skill, clinical decision‐making power and as a result identifying patient needs and selecting the best nursing practices. This can directly influence the patient's safety and improvement (Pitt et al., 2014, 2015).

Applicants understood the status of nursing with high job security and, at the same time, with limited job growth and advancement. The reason for choosing this field was also attributed to the early employment. This finding is consistent with the previous studies. In these studies, the entry of people into nursing is not based on their enthusiasm, and it has been reported only due to job security (Glerean et al., 2017; Mkala, 2013; Tayebi et al., 2013); this could reduce the quality of nursing care (Swarna, 2015). This problem is due to the opportunity of faster employment of graduates of this field than other fields in Iran (Shamsi & Peyravi, 2020).

Based on the results of this study, applicants' perception of the nursing profession is influenced by the media, academic–career counsellors, personal factors and family and friends. The effects of the media on people's perception of nursing are recognized and it has a lot of influence on the recruitment of new nurses (Weaver et al., 2013). The media has adopted the stereotyped methods in introducing nursing and it has not changed that much in recent years (Koo & Lin, 2016; Weaver et al., 2013). The impact of the media on the perception of nursing applicants may be enormous because the current generation is more exposed to the media than any other generation (Shatto & Erwin, 2016). The role of family and friends in developing the applicants' perception of the profession is evident in the review of literature (Glerean et al., 2017, 2019). In addition, the influence of teachers and career counsellors in choosing a job and understanding nursing has already been identified in the literature (Neilson & McNally, 2013; Önder et al., 2014). The results of this study showed that the information that career counsellors give to young people about the nursing profession is negative. These negative perceptions could result in fewer nursing applicants.

## 5 CONCLUSION AND IMPLICATIONS

The results of this study showed that applicants generally enter nursing education with old and traditional understanding. Research should focus on planning interventions to change the public image of nursing. To prevent misconceptions in career guidance, accurate and realistic information should be provided about the actual work of nursing concerning nurses' various roles and duties, the knowledge and skills necessary to enter the field. Nursing schools can be a key factor in creating an accurate and positive image of the nursing profession. Emphasis should also be placed on the close collaboration among the nursing schools, career counsellors and high schools. Video clips related to a nurse's real work, developing websites managed by nurses to share more information, and the opportunity of visiting the nursing schools by high school students could promote a positive and realistic image of nursing in the community. This could influence the perception of the nursing applicants. Research should also focus on developing strategies to recruit and retain the motivated nursing students who have realistic perceptions of the nursing profession.

## 6 LIMITATIONS

Data collection were carried out only in three provinces of western Iran. Hence, perceptions of the nursing applicants in other cities and outside Iran may be different. Also, the social and cultural conditions of the society can affect the findings of this study. The findings of this study are related to the context of Iran. Therefore, it is necessary to conduct this study in the context of other societies.

## AUTHOR CONTRIBUTIONS

Study conception and design: VZ, AG, LV and FB. Data collection: VZ and FB. Data analysis and interpretation: All authors. Drafting of the article: All authors. Critical revision of the article: All authors.

## FUNDING INFORMATION

The financial resources of this study have been provided by Tabriz University of Medical Sciences.

## CONFLICT OF INTEREST

None of the authors had a conflict of interest.

## ETHICAL APPROVAL

This study is part of the results of an approved dissertation of Ph.D. in Nursing at the Tabriz University of Medical Sciences, which has been confirmed by the ethics committee of the university (Ethical code: IR.TBZMED.REC.1397.583). Before starting the study, the consent of the managers of the sampling centres was obtained. After selecting the participants, the study's objectives were explained to them at the beginning of the interview, and oral and written consents were obtained from them to participate in the study and for the audio recording. Participants were assured that the recorded material would be used without mentioning their names or details. Participants had the right to withdraw at any stage of the research.

## Data Availability

The data sets used during this study are available from the corresponding author on reasonable request.
